# Supplementary material for: Inferring extrinsic noise from single-cell gene expression data using approximate Bayesian computation
Source: BMC Syst Biol. 2016 Aug 22;10(1):81. doi: 10.1186/s12918-016-0324-x (PMC4994381; doi:10.1186/s12918-016-0324-x)
Supplement: Additional file 1 — Derivation of the Poissonian relationship between the number of surviving protein molecules and mRNA lifetime. (PDF 146 kb) [file 12918_2016_324_MOESM1_ESM.pdf]

# DERIVATION OF THE POISSONIAN RELATIONSHIP BETWEEN THE NUMBER OF SURVIVING PROTEIN MOLECULES AND MRNA LIFETIME

OLEG LENIVE, PAUL D.W. KIRK, & MICHAEL P.H. STUMPF

Derivation of the Poissonian relationship between the number of surviving protein molecules and mRNA lifetime

## 1. Derivations

Consider the following system of reactions.

$$\emptyset \xrightarrow{\lambda} X$$

$$X \xrightarrow{\rho} Y$$

$$X \xrightarrow{\delta} \emptyset$$

$$Y \xrightarrow{\gamma} \emptyset$$

The system is allowed to run, starting with zero tokens of $X$ and $Y$, from time $t_0$ until time $t_m$. The following table lists events of interest which can occur in the system and the symbols used to represent the number of times each even occurs in the time interval $T = t_m - t_0$.

| Symbol | Event being counted |
|---|---|
| $i$ | $X$ is produced |
| $j$ | $X$ survived until time $t_m$ |
| $\hat{i}$ | $X$ does not survive until time $t_m$ (i.e. $X$ is degraded or converted to $Y$) |
| $\hat{j}$ | $X$ is degraded |
| $k$ | $X$ is converted into $Y$ |
| $l$ | $Y$ survives until time $t_m$ |

### 1.1. Deriving probability distributions for the numbers of events.

A standard result is that the number of events which occur over a time period will have a Poisson distribution if the events are occurring with a uniform probability in a time interval. This applies to the production of $X$.

$$P(i) = \frac{(\lambda T)^i}{i!} e^{-\lambda T} \tag{1}$$

Where $P(i)$ is the probability that $X$ is produced $i$ times during the time period $T$.

1.2. **Finding the number of surviving tokens of $X$.** In order to find the probability distribution of the number of $X$ tokens after the time period $T$ we consider the probability that $j$ tokens of $X$ remain given that $i$ tokens of $X$ were produced.

$$P(j|i) = \binom{i}{j} p^j (1-p)^{i-j} \tag{2}$$

Where $p$ is the probability that $X$ survives given that it was produced during the time period $T$.

Let $P(j)$ be the probability that $j$ tokens of $X$ were produced during $T$ and survived regardless of the number $(i)$ of $X$ tokens produced.

$$P(j) = \sum_{i=j}^{\infty} P(i)P(j|i) \tag{3}$$

Using equations 1 and 2.

$$\begin{aligned} P(j) &= \sum_{i=j}^{\infty} \frac{(\lambda T)^i}{i!} e^{-\lambda T} \binom{i}{j} p^j (1-p)^{i-j} \\ &= e^{-\lambda T} p^j \sum_{i=j}^{\infty} \frac{(\lambda T)^i}{i!} \frac{i!}{j!(i-j)!} p^j (1-p)^{i-j} \\ &= \frac{e^{-\lambda T} p^j}{j!(1-p)^j} \sum_{i=j}^{\infty} \frac{q^i}{(i-j)!} \end{aligned}$$

Where $q = \lambda T(1-p)$.
Considering just the summation term.

$$\begin{aligned} \sum_{i=j}^{\infty} \frac{q^i}{(i-j)!} &= \frac{q^j}{0!} + \frac{q^{j+1}}{1!} + \frac{q^{j+2}}{2!} + \frac{q^{j+2}}{2!} + ... + \frac{j+i}{(i-j)!} + ... \\ &= q^j \left(1 + \frac{q}{1} + \frac{q^2}{2!} + \frac{q^3}{3!} + ... + \frac{q^i}{(i-j)!} + ...\right) \\ &= q^j \sum_{n=0}^{\infty} \frac{q^n}{n!} = q^j e^q \\ &= (\lambda T)^j (1-p)^j e^{\lambda T(1-p)} \end{aligned}$$

Thus, the expression for $P(j)$ can be written as:

$$P(j) = \frac{e^{-r} p^j (\lambda T)^j (1-p)^j e^{\lambda T(1-p)}}{j!(1-p)^j}$$

(4)
$$P(j) = \frac{(p\lambda T)^j}{j!} e^{-p\lambda T}$$

$P(j)$ has a Poisson distribution with the parameter $p\lambda T$.

Next, we need to find $p$. The probability that $X$ was produced during a infinitesimally short time interval $dt$ is given by $dt/T$. That is to say that $X$ is produced once at some point during $T$ and the probability of $X$ production is uniform over $T$. The probability of a token of $X$ produced at time $t$ surviving until time $t_m$ follows an exponential decay with a rate $\delta'$. In this case $\delta' = \rho + \delta$. Thus the probability of a token of $X$ being produced at time $t$ and surviving until $t_m$ is given by

$$\frac{dt}{T} e^{-\delta'(t_m - t)}$$

The overall probability that $X$ survives given that it was produced can be found by integrating over the time period $T$.

$$p = \int_{t_0}^{t_m} \frac{dt}{T} e^{-\delta'(t_m - t)}$$

(5)
$$p = \frac{1}{\delta' T} \left(1 - e^{-\delta' T}\right)$$

Where $T = t_m - t_0$.

The numbers of the other events are also Poisson distributed. Similarly to $P(j)$, the number of tokens of $X$ which did not survive ($P(\hat{i})$) will also be Poisson distributed.

$$P(\hat{i}) = \frac{p_{\hat{i}} \lambda T}{\hat{i}!} e^{-p_{\hat{i}} \lambda T}$$

Where $p_{\hat{i}} = (1-p) = 1 - \frac{1}{\delta' T} \left(1 - e^{\delta' T}\right)$.

Also, for $P(k)$, the distribution of the number of tokens of $Y$ produced

$$P(k) = \frac{(p_k \lambda T)^k}{k!} e^{-p_k \lambda T}$$

with $p_k = \frac{\rho}{\rho + \delta}(1-p)$.
