# Supplementary figures and images for: Inferring extrinsic noise from single-cell gene expression data using approximate Bayesian computation
Source: BMC Syst Biol. 2016 Aug 22;10(1):81. doi: 10.1186/s12918-016-0324-x (PMC4994381; doi:10.1186/s12918-016-0324-x)

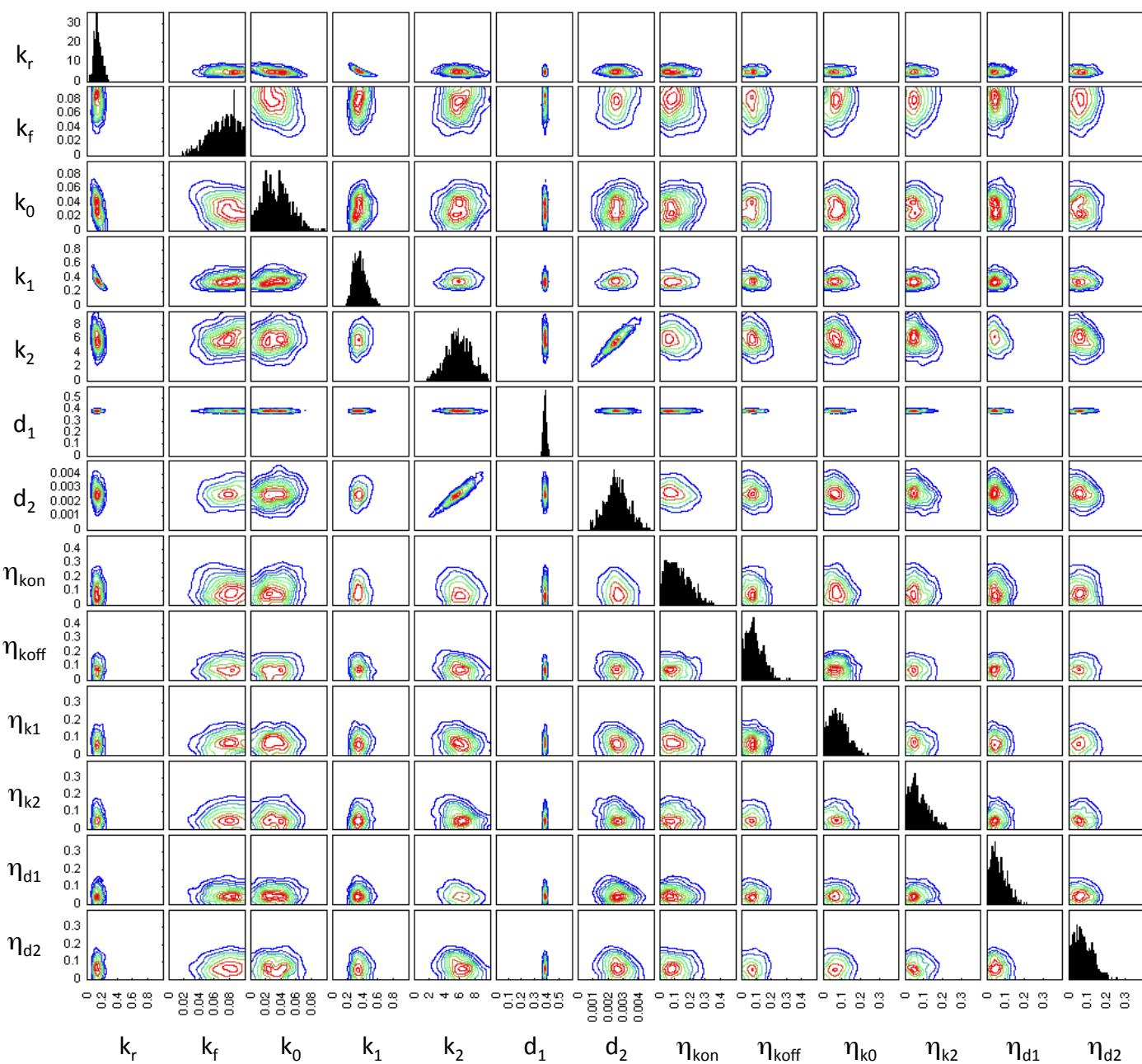

Supplement: Additional file 2 — Parameter posteriors for the expression model of the rcsB gene. (PDF 180 kb) [file 12918_2016_324_MOESM2_ESM.pdf]

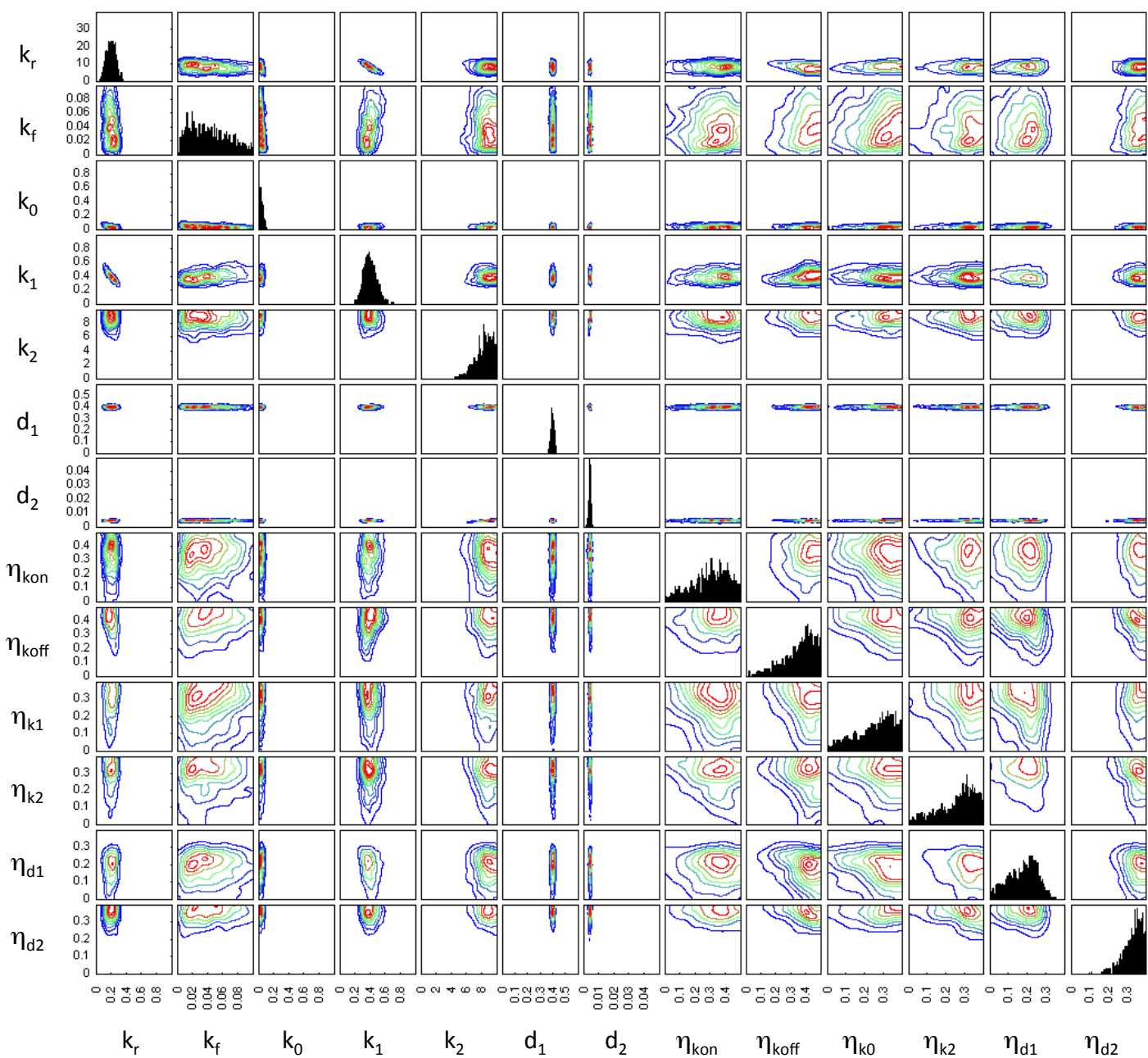

Supplement: Additional file 3 — Parameter posteriors for the expression model of the yiiU gene. (PDF 179 kb) [file 12918_2016_324_MOESM3_ESM.pdf]

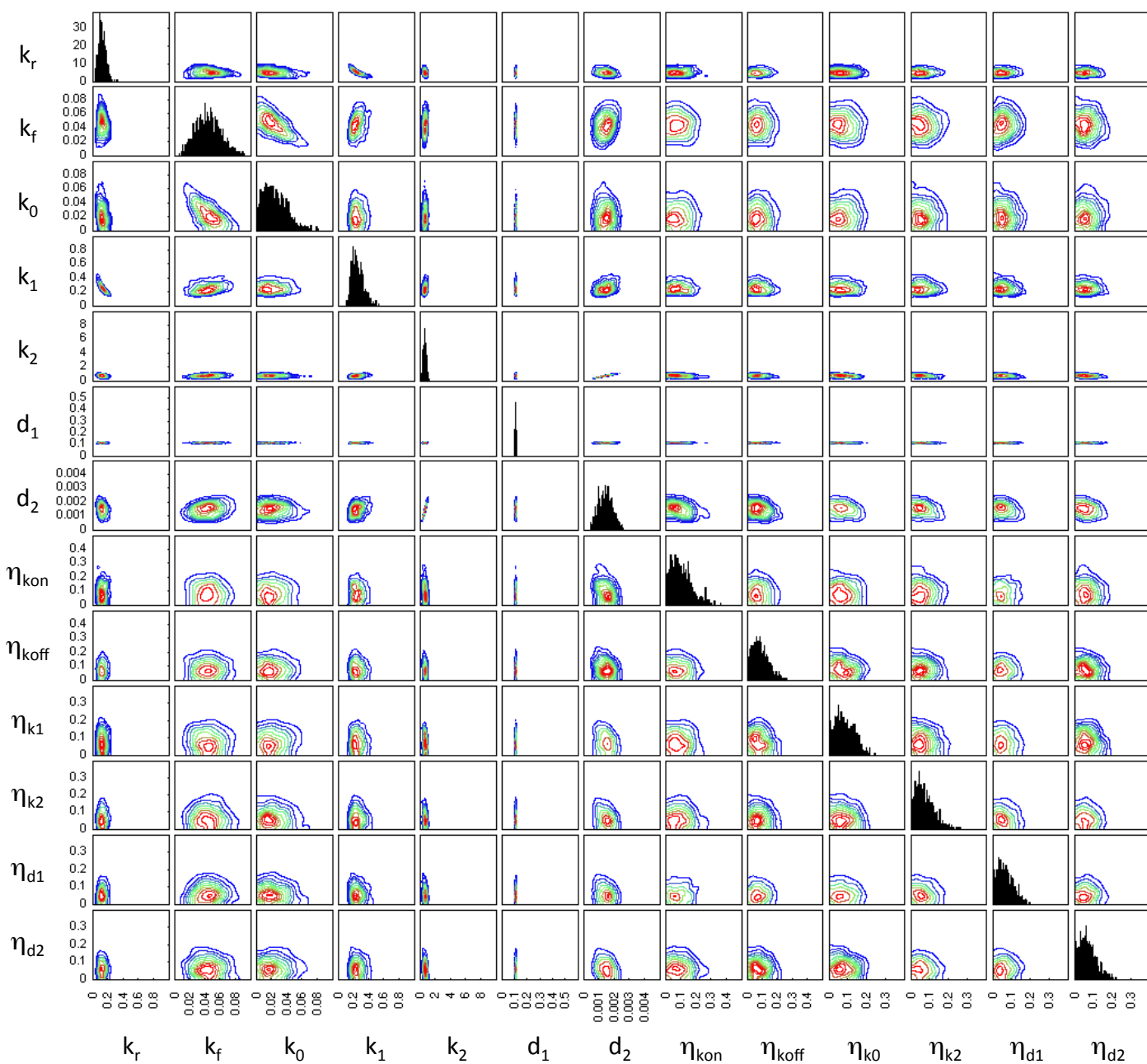

Supplement: Additional file 4 — Parameter posteriors for the expression model of the yebC gene. (PDF 160 kb) [file 12918_2016_324_MOESM4_ESM.pdf]

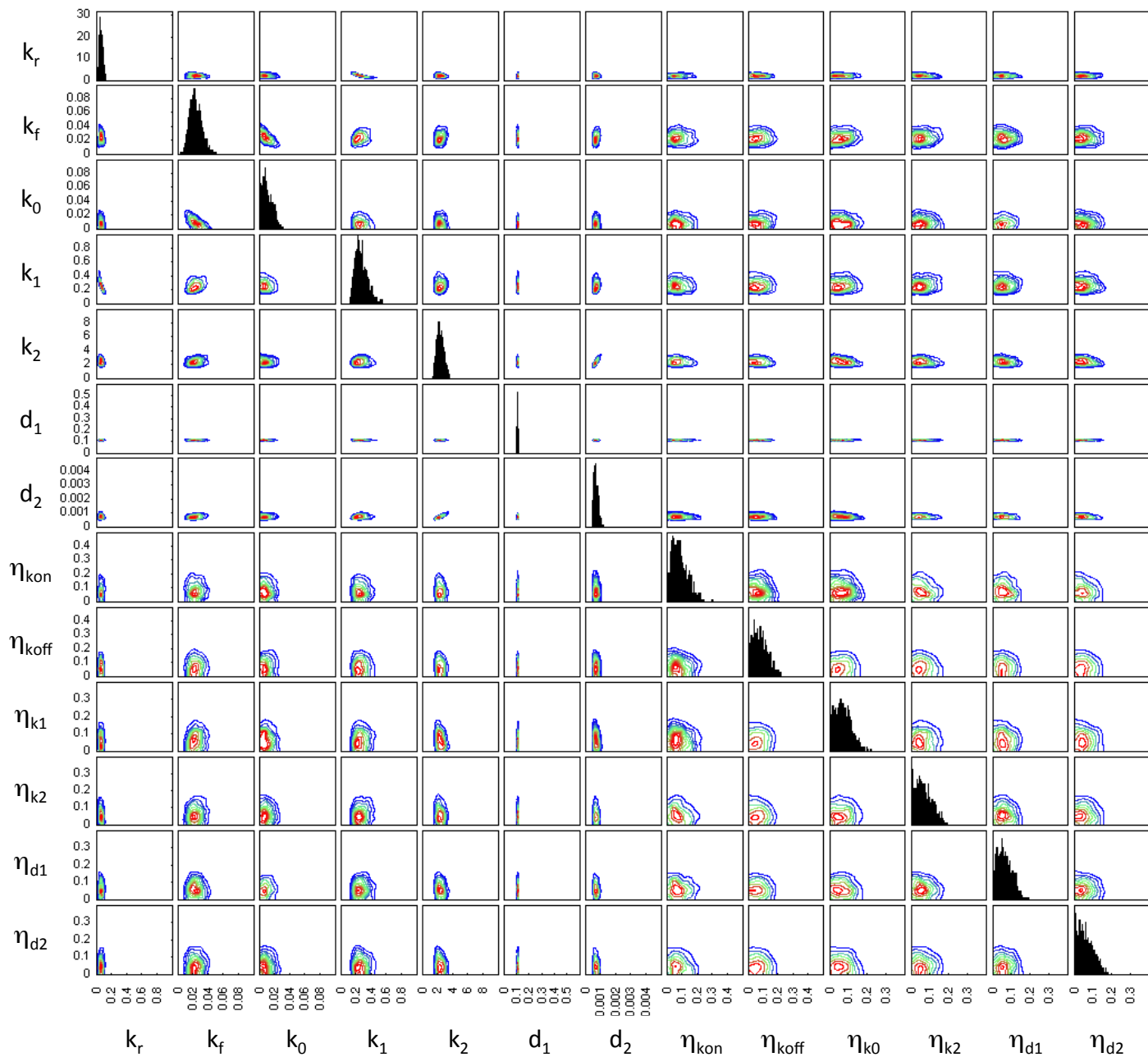

Supplement: Additional file 5 — Parameter posteriors for the expression model of the eno gene. (PDF 138 kb) [file 12918_2016_324_MOESM5_ESM.pdf]
